# High frequency of antiviral drug resistance and non-b subtypes in HIV-1 patients failing antiviral therapy in Cuba

**DOI:** 10.7448/IAS.17.4.19754

**Published:** 2014-11-02

**Authors:** Vivian Kouri, Yoan Alemán, Lissette Pérez, Jorge Pérez, Carlos Fonseca, Consuelo Correa, Carlos Aragonés, Jorge Campos, Delmis Álvarez, Yoeri Schrooten, Lore Vinken, Celia Limia, Yudira Soto, Anne-Mieke Vandamme, Kristel Van Laethem

**Affiliations:** 1 Virology Department, Laboratory of Sexually Transmitted Diseases, Institute of Tropical Medicine “Pedro Kourí”, Havana, Cuba; 2 Institute of Tropical Medicine “Pedro Kourí”, Havana, Cuba; 3 Institute of Tropical Medicine “Pedro Kourí”, Hospital Division, Havana, Cuba; 4 Computer Department, Institute of Tropical Medicine “Pedro Kourí”, Havana, Cuba; 5 Department of Microbiology and Immunology, Laboratory for Clinical and Epidemiological Virology, Rega Institute for Medical Research, KU Leuven, Leuven, Belgium; 6 Department of Microbiology and Immunology, Laboratory for Clinical and Epidemiological Virology, Rega Institute for Medical Research and Centro de Malária e Outras Doenças, Leuven, Belgium

## Abstract

**Introduction:**

Emergence of HIV-1 drug resistance may limit the sustained benefits of antiretroviral therapy (ART) in settings with limited laboratory monitoring and drug options. The objective is to implement the surveillance of drug resistance and subtypes in HIV-1 patients failing ART in Cuba.

**Methods:**

This study compiled clinical and genotypic drug resistance data 588 ART-experienced HIV-1 patients attending a clinical center in Havana in 2009–2013. Drug resistance testing was performed as part of routine clinical care. Drug resistance mutations and levels were determined using Rega version 8.0.2.

**Results:**

Eighty-three percent received solely ART containing at least three drugs. Patients from 2009 to 2010 were longer treated (median: 4.9 vs 2.7 years) and exposed to more ART regimens (median: 4 vs 2 regimens) compared to patients from 2011–2013. Nucleoside reverse transcriptase inhibitor (NRTI), non-nucleoside RTI (NNRTI) and PI mutations were present in 83.5, 77.4 and 52.0%. Full-class resistance (FCR) to NRTI, NNRTI, PI and multidrug resistance (MDR) were detected in 25.0, 33.7, 11.4 and 6.3%. FCR to NRTI, NNRTI, PI and MDR were present in 12.8, 28.7, 0 and 0% after first-line failure (164 patients) and in 23.1, 34.6, 3.8 and 3.1% after second-line failure (130 patients). Subtype B (32.5%), BG recombinants (19.6%) and CRF19_cpx (16.2%) were the most prevalent genetic forms. Subtype distribution did not change significantly between 2009–2010 and 2011–2013, except for BG recombinants that increased from 12.2 to 21.3% (p=0.002).

**Conclusions:**

Our study found a high prevalence of drug resistance and supports the need for appropriate laboratory monitoring in clinical practice and access to drug options in case of virological failure.

